# Effects of methamphetamine abuse on spatial cognitive function

**DOI:** 10.1038/s41598-018-23828-y

**Published:** 2018-04-03

**Authors:** Yan-Lin Luo, Jing-Wei Bian, Zhi-Jun Zheng, Li Zhao, Song Han, Xiao-Hong Sun, Jun-Fa Li, Guo-Xin Ni

**Affiliations:** 1 Department of Neurobiology, Capital Medical University, Beijing, China; 2 Shanghai Compulsory Isolation Detox Center, Shanghai, China; 3 Department of Rehabilitation Medicine, First Affiliated Hospital, Fujian Medical University, Fuzhou, China

## Abstract

Methamphetamine (MA) abuse has been rising rapidly over the past decade, however, its impact in spatial cognitive function remains unknown. To understand its effect on visuospatial ability and spatial orientation ability, 40 MA users and 40 non-MA users conducted the Simple Reaction Task (Task 1), the Spatial Orientation Task (Task 2), and the Mental Rotation Task (Task 3), respectively. There was no significant difference in either accuracy or reaction time (RT) between 2 groups in Task 1. During Task 2, in comparison with non-MA users, MA users performed poorer on RT, but not in accuracy for foot and hand stimuli. In addition, both non-MA and MA users responded much more quickly to upward stimuli than downward stimuli on vertical surface, however, only non-MA users exhibited leftward visual field advantage in horizontal orientation processing. As for Task 3, MA users exhibited poorer performance and more errors than their healthy counterparts. For each group, linear relationship was revealed between RT and orientation angle, whereas MA abuse led to longer intercept for all stimuli involved. Our findings suggested that MA abuse may lead to a general deficit in the visuospatial ability and the spatial orientation ability with more serious impact in the former.

## Introduction

Methamphetamine (MA) is the derivative of cathinone found naturally in the leaves of the khat plant (Catha edulis) which has been chewed for centuries in some Middle Eastern and African countries for its amphetamine-like euphoric effects^1^. MA abuse has been rising rapidly over the past decade, and therefore regarded as a public health problem, both nationally and globally. Numerous studies have been conducted to understand its negative impacts in a variety of aspects. For instances, it has been linked to such psychological problems as depression, anxiety, social isolation, psychosis, bipolar and disorder^2–7^. Everyday functioning problems have also be demonstrated in interpersonal difficulties^8,9^, impulsivity^10,11^, and unemployment^12^. Moreover, considerably negative effects have been reported on many neurocognitive functions, including attention^13^, memory, learning^14^, executive function, and fine motor speed^15,16^.

Spatial ability is the individual ability to deal with objects in space mentally, and plays an important role in daily life. Unfortunately, our current understanding is rather limited about the impact of MA abuse in spatial processing. A number of small animal studies suggest that MA exposure may lead to significant deficit in spatial learning^17–20^, and poor performances in spatial task^21^. It was also reported that abstinent MA users for 6 months presented considerably impaired social emotion cognition and spatial working memory^22^. However, the impact of MA abuse has been rarely investigated in human spatial cognitive function. Notably, accumulating evidence indicates that 3,4-methylenedioxymethamphetamine (MDMA) is associated with the impairment of visuospatial processing. Being ring-substituted derivatives of amphetamine (AMTH), MDMA and MA belong to the same type of psycholoactive drug and share a similar mechanism of action^23,24^. Using the Tile Manipulation Test, MDMA was reported to induce deficit in spatial working memory and planning^25^. In addition, MDMA users presented impaired processing of visuospatial information during the Spatial Working Memory Span and the Simple Spatial Span Task, with worse performance during the former task (under high cognitive loading)^26^. A recent systemic review demonstrated that MDMA may negatively affect user’s performances in a range of visuospatial tasks, whereas controversy existed on the task of judgment of visual-spatial stimulus characteristics^27^.

It is therefore hypothesized that, similar to MDMA, MA abuse may impair the spatial processing. Since spatial ability can be measured by spatial perception, mental rotation, and spatial visualization^28–30^, this work was designed to examine both the spatial orientation ability and the visuospatial ability in MA abusers and non-MA users using the Spatial Orientation Task and the Mental Rotation Task, respectively. As the basis of spatial recognition function, the spatial orientation task is much easier to be performed than the visuospatial task. In this regard, negative effect of MA abuse on spatial orientation ability may be either not apparent, or less serious than the visuospatial ability. Hence, the second hypothesis of this work is that MA abuse may mainly affect abusers’ visuospatial ability, rather than the spatial orientation ability.

## Results

### Demographics and clinical characteristics in two groups

The demographics and clinical characteristics of MA (MA+) and non-MA (MA−) users were shown in Table 1. Significant difference did not exist between two groups in age, educational attainment, Edinburgh handedness scale, mini mental state examination (MMSE), state-anxiety/trait-anxiety scale, and withdrawal syndrome scale of craving, respectively.

**Table 1:** Demographics and clinical data in MA+ and MA− groups.

	MA− group	MA+ group	T value	P value
Age (years old)	37.50 ± 1.37	35.40 ± 1.53	1.110	0.271
Education levels (years)	9.90 ± 0.53	9.70 ± 0.48	0.387	0.700
Edinburgh handedness scale	93.90 ± 1.51	95.60 ± 1.38	−0.887	0.378
MMSE	28.90 ± 0.21	29.10 ± 0.18	−0.752	0.455
State-anxiety	42.70 ± 1.09	44.20 ± 1.02	−1.061	0.292
Trait-anxiety	43.90 ± 1.27	41.80 ± 1.09	1.251	0.215
Age at onset (years old)		27.70 ± 1.37		
Usage amount at onset (g/day)		0.14 ± 0.02		
The most amount of use (g/day)		1.30 ± 0.11		
Duration of use (months)		26.60 ± 3.13		
Withdrawal syndrome scale of craving		23.00 ± 0.23		

### Effect of MA on simple reaction task (Task 1)

**Figure 1:**
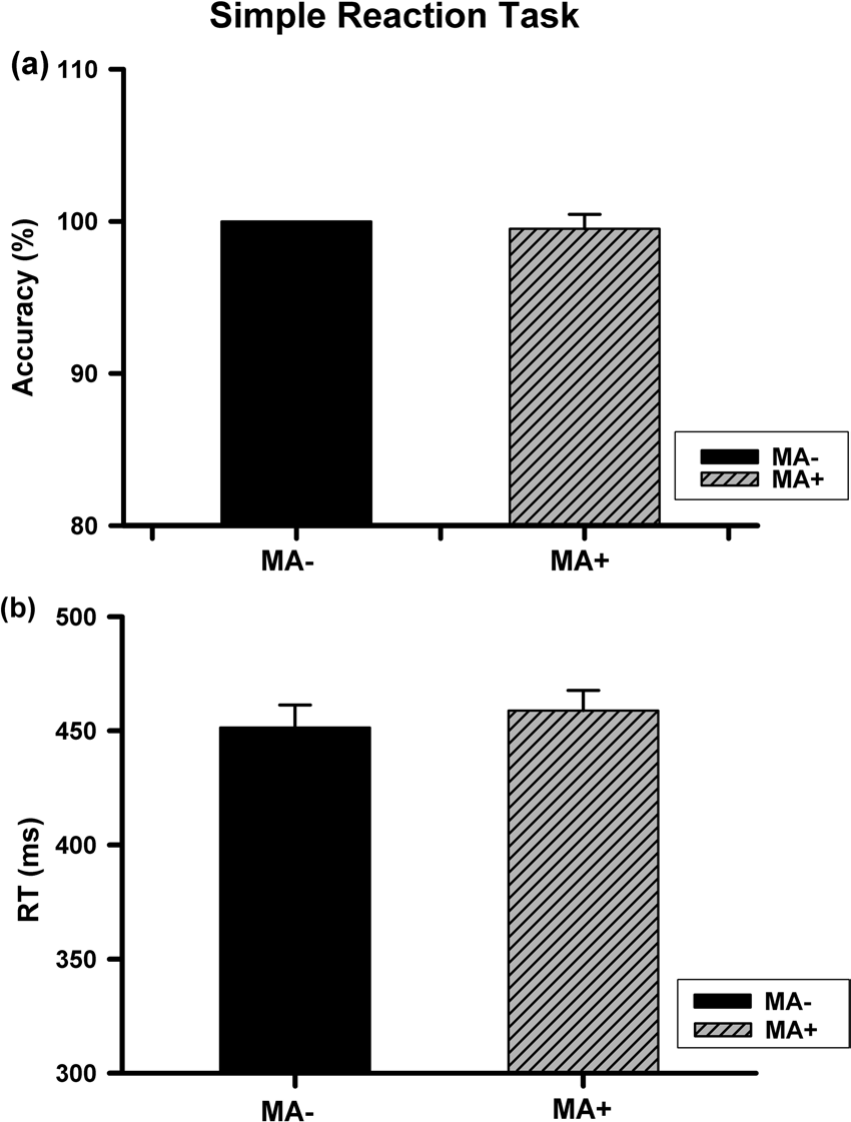
Accuracy and reaction time in the simple reaction task in MA− and MA+ groups. No significant difference was revealed between two groups in either accuracy (**a**) or RT (**b**).

In this study, simple reaction task was conducted to understand if there is a general deficit in attention or motor ability between MA+ and MA− groups. During this task, the basic motor speed and motor ability were measured by discriminating hand or foot pictures. Table 2 showed the results of accuracy and reaction time (RT) in two groups. By independent samples t tests analysis, significant difference was not observed between two groups in either accuracy (t = 0.997, p = 0.322) or RT (t = 0.560, p = 0.577) (Fig. 1, n = 38 per group), indicating that MA abuse did not cause a significant deficit in either general attention or motor ability. In other words, a general RT slowing and reduction in accuracy reported below should be caused by a deficit in spatial ability.

**Table 2:** Accuracy and RT at leftward and rightward orientation for foot stimuli, as well as upward and downward orientation for hand stimuli on spatial orientation task in two groups.

	Foot		Hand	
	Rightward	Leftward	Upward	Downward
**Accuracy (%)**				
MA−	98.7 ± 0.2	98.4 ± 0.3	99.0 ± 0.4	97.8 ± 0.4
MA+	97.0 ± 0.5	96.5 ± 1.0	98.4 ± 0.4	97.1 ± 0.7
**RT (ms)**				
MA−	602 ± 11	570 ± 13^a^	557 ± 10^b^	579 ± 10
MA+	630 ± 16	630 ± 21	577 ± 12^b^	617 ± 12

^a^p < 0.05, foot leftward stimulus vs. rightward stimulus for MA− group;

^b^p < 0.05, hand upward stimulus vs. downward stimulus for both MA+ and MA− groups.

### Effect of MA on spatial orientation ability (Task 2)

**Figure 2:**
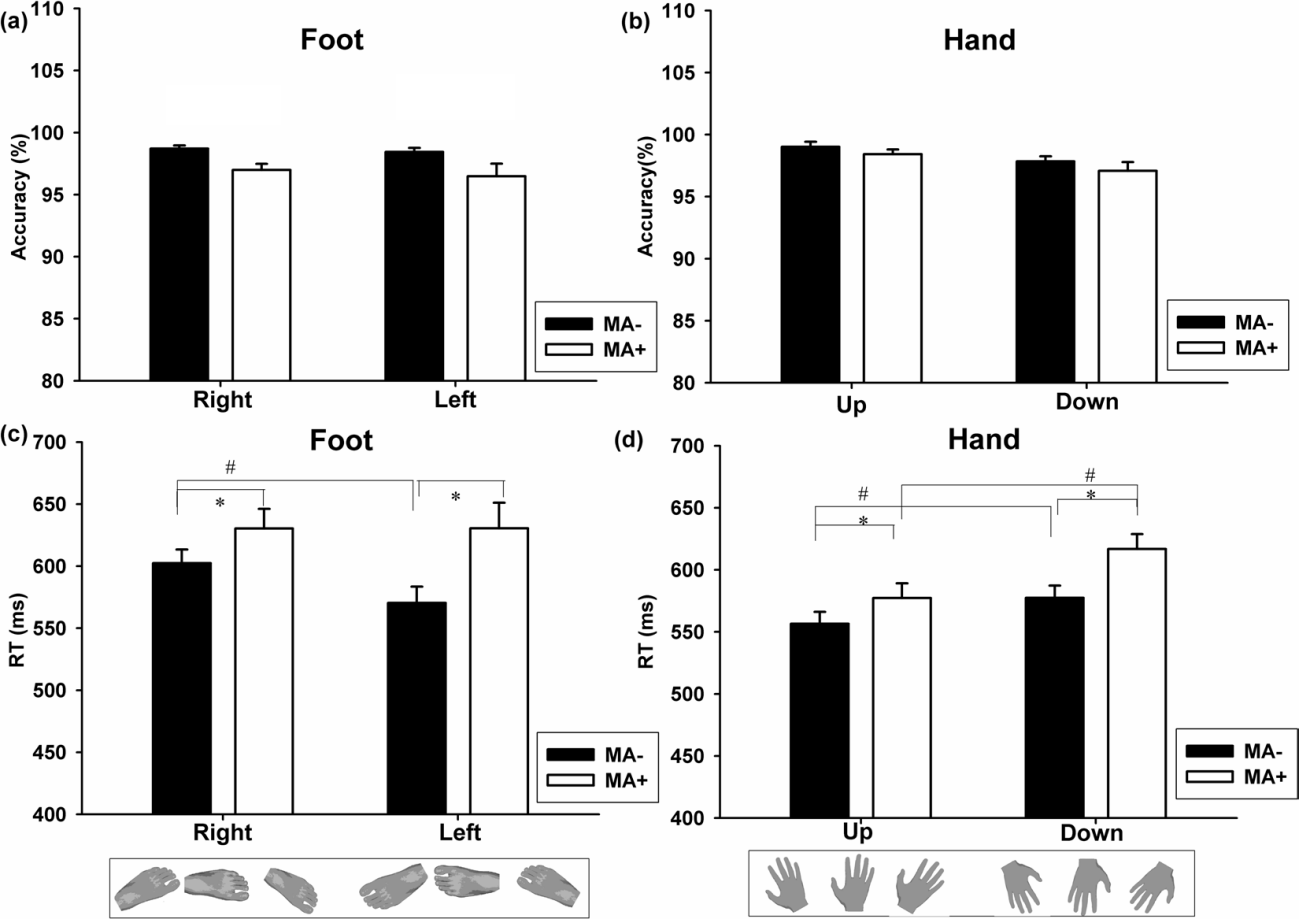
Accuracy and reaction time at each orientation angle for both foot and hand stimuli in MA− and MA+ groups. Foot stimuli were oriented either leftward (240°/270°/300°) or rightward (60°/90°/120°) (**a**,**c**), whereas hand stimuli either upward (330°/0°/30°) or downward (150°/180°/210°) (**b**,**d**). In comparison with non-MA abusers, MA abusers reacted with lower accuracy for foot stimuli, and slower speed for either foot or hand stimuli. Furthermore, non-MA abusers but not MA abusers respond more quickly to leftward foot stimuli than rightward foot ones (**c**). All participants reacted more quickly to upward hand stimuli than downward hand stimuli (**d**). *p < 0.05, MA+ group vs. MA− group; ^#^p < 0.05, leftward vs. rightward for foot stimuli, or upward vs. downward for hand stimuli.

Spatial orientation ability is the basis of spatial ability. As such, Task 2 was conducted to evaluate such ability in MA+ and MA− groups by judging the direction of single hand (thumb) or foot (toe) picture. The corresponding accuracy and RT were calculated at leftward and rightward orientation for foot stimuli, as well as upward and downward orientation for hand stimuli in MA+ and MA− groups. Afterwards, these data were further analyzed by repeated two-way ANOVA for within-subject factor (2 levels of orientation angle: left or right for foot; up or down for hand), and between-subject factor (2 groups: MA+ and MA− group). As for the accuracy for either foot or hand stimuli, no significant main effect was found for each factor (angle or group) or for the interaction between them {For foot: group [F(1, 37) = 2.15, p = 0.190, η2 = 0.02], orientation angle [F(1, 37) = 0.764, p = 0.388, η2 = 0.003], and interaction [F(1, 37) = 0.077, p = 0.783, η2 < 0.01]; For hand: group [F(1, 37) = 1.313, p = 0.259, η2 = 0.013], orientation angle [F(1, 37) = 1.623, p = 0.143, η2 < 0.01], and interaction [F(1, 37) = 0.049, p = 0.827, η2 < 0.01]} (Fig. 2a,b and Table 2).

The results of RT suggested significant main effect for factor group [F(1, 37) = 4.628, p = 0.038, η2 = 0.05], orientation angle [F(1, 37) = 6.924, p = 0.012, η2 = 0.006] and their interaction [F(1, 37) = 6.058, p = 0.019, η2 = 0.007] for foot stimuli on horizontal surface, respectively (Fig. 2c). Similar results were observed for factor group [F(1, 37) = 4.453, p = 0.042, η2 = 0.046] and orientation angle [F(1, 37) = 46.437, p < 0.001, η2 = 0.047], but NOT for their interaction [F(1, 37) = 3.711, p = 0.062, η2 = 0.004], for hand stimuli on vertical surface (Fig. 2c,d). A post-hoc analysis (Bonferroni) on horizontal surface indicated that leftward bias existed only in non-MA users, who responded much more quickly to leftward stimuli than rightward stimuli (t = 3.594, p < 0.001). The statistically significant interaction between orientation angle and group for foot stimuli would lend evidence towards our finding that leftward bias existed only in non-MA users, which may be attributable to differences between 2 groups in RT to the leftward foot  stimuli (t = 2.798, p = 0.008)(Fig. 2d). On vertical surface, however, both MA and non-MA users responded much more quickly to upward stimuli than to downward stimuli (Fig. 2c,d and Table 2). The results of Task 2 indicated that MA abuse may induce a deficit in spatial orientation ability, mainly on horizontal surface.

### Effect of MA on visuospatial ability (Task 3)

**Figure 3:**
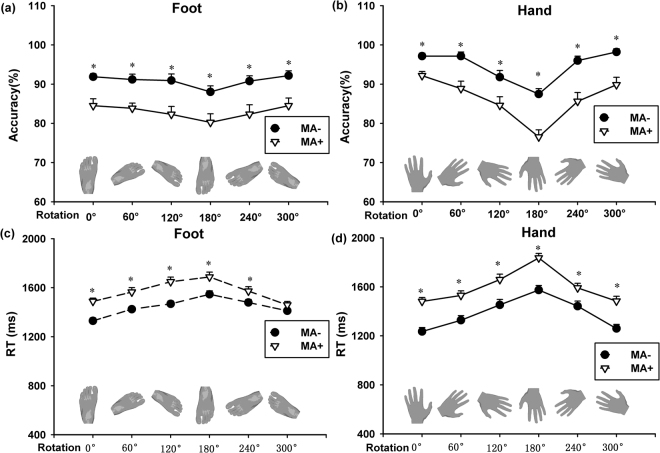
Accuracy and reaction time at each orientation on both hand and foot laterality tasks in MA- and MA+ groups. During the tasks, both foot and hand stimuli were oriented at 0°, 60°, 120°, 180°, 240°, and 300°, respectively. Significantly lower accuracy was found in MA+ group than MA− group at each orientation angle for either foot (**a**) or hand stimuli (**b**). As for reaction time, MA+ group preformed poorer than MA− group for foot stimuli at each orientation (with exception of 300°) (**c**) and for hand stimuli (**d**). *p < 0.05, MA+ group vs. MA− group.

Visuospatial ability is a critical aspect of cognitive function. Task 3 was conducted to assess this ability in MA+ and MA− groups by judging the lateral of single hand or foot stimulus. The accuracy and RT were analyzed by repeated two-way ANOVA for within-subject factor (6 levels of orientation angle: 0°, 60°, 120°, 180°, 240°, and 300° for either hand or foot), and between-subject factor (2 groups: MA+ and MA− group). For foot stimuli, significant main effects were observed in the accuracy for factor group [F(1, 37) = 38.395, p < 0.001, η2 = 0.126], but NOT for orientation angle [F(5, 185) = 2.249, p = 0.051, η2 = 0.016] and their interaction [F(5, 185) = 0.0642, p = 0.997, η2 < 0.001]. As for hand stimuli, however, significant main effects were found in the accuracy for factor group [F(1, 37) = 28.567, p < 0.001, η2 = 0.133], and orientation angle [F(5, 185) = 26.451, p < 0.001, η2 = 0.145], but NOT for their interaction [F(5, 185) = 1.302, p = 0.265, η2 = 0.007]. Post-hoc analysis (Bonferroni) revealed that MA+ group exhibited poorer performance than MA− group at each orientation angle (p < 0.05) for either foot stimuli (Fig. 3a) or hand stimuli (Fig. 3b and Table 3).

**Table 3:** Accuracy and RT at each orientation for hand and foot stimuli on mental rotation task in two groups.

	Accuracy (%)						RT (ms)					
	0°	60°	120°	180°	240°	300°	0°	60°	120°	180°	240°	300°
**Foot**												
MA−	91.9 ± 0.8	91.2 ± 1.4	90.9 ± 1.6	88.0 ± 1.5	90.8 ± 1.4	92.2 ± 1.2	1330 ± 24	1425 ± 19	1468 ± 24	1546 ± 29	1480 ± 25	1412 ± 18
MA+	84.5 ± 1.8^a^	83.8 ± 1.3^a^	82.3 ± 2.0^a^	80.2 ± 2.2^a^	82.3 ± 2.4^a^	84.5 ± 1.9^a^	1489 ± 27^b^	1565 ± 37^b^	1649 ± 38^b^	1688 ± 40^b^	1573 ± 36^b^	1459 ± 29^b^
**Hand**												
MA−	97.1 ± 0.8	97.1 ± 1.1	91.8 ± 1.7	87.5 ± 1.4	96.0 ± 1.1	98.2 ± 0.8	1235 ± 32	1327 ± 37	1453 ± 45	1574 ± 37	1443 ± 42	1260 ± 34
MA+	92.2 ± 1.1^a^	88.9 ± 1.9^a^	84.6 ± 2.2^a^	76.5 ± 1.8^a^	85.6 ± 2.3^a^	89.8 ± 1.9^a^	1481 ± 32^b^	1530 ± 38^b^	1659 ± 44^b^	1836 ± 38^b^	1592 ± 38^b^	1484 ± 39^b^

^a^p < 0.05, MA+ group vs. MA− group for foot and hand stimulus for accuracy.

^b^p < 0.05, MA+ group vs. MA− group for foot and hand stimulus for RT.

Similarly, the results of RT demonstrated significant main effects for factor group [F(1, 37) = 17.855, p < 0.001, η2 = 0.10], orientation angle [F(5, 185) = 24.320, p < 0.001, η2 = 0.119] and their interaction [F(5, 185) = 2.480, p = 0.033, η2 = 0.011] for foot stimuli, respectively. However, significant main effects were found for group [F(1, 37) = 17.597, p < 0.001, η2 = 0.145] and orientation angle [F(5, 185) = 71.626, p < 0.001, η2 = 0.180], but NOT for interaction of the two factors [F(1, 37) = 1.484, p = 0.197, η2 = 0.004] for hand stimuli. Post-hoc analysis suggested that MA+ group performed worse than MA- group at each orientation angle (p < 0.05) for foot stimuli (except 300°) (Fig. 3c) and hand stimuli (Fig. 3d and Table 3).

During the mental rotation task, the transformation of visuospatial mental image is crucial to action, navigation and reasoning^31^. In order to determine the correlations of deficit in spatial recognition with the duration of MA use, a linear regression equation of RT was further calculated in this study between mental rotation time and angular disparity. Within this equation, the slope of linear regression equation means the averaged change associated with rotating an object for an additional degree, while the intercept reflects the contribution of non-rotational processes. What is more, linear relationship between mental rotation time and angular disparity could be used as an indication to determine either an object-based strategy or egocentric-based strategy^31–35^. Both strategies are involved in motor image, motor plan and spatial recognition which are important for daily life. In this regard, MA+ group was further assigned into short-term [n = 16, 9 males and 7 females, duration for 0.5–1.5 years (0.88 ± 0.34)] and long-term [n = 22, 11 males and 11 females, duration for 2–7 years (3.1 ± 0.29)] sub-groups. RTs at different orientation angles from 0° to 180° were then computed after combining data of 0° with 360°, 60° with 300°, and 120° with 240° in each group to obtain the corresponding slope and intercept of linear regression equation with orientation angle. In these three groups, significant linear relationship was revealed between RT and orientation angle. Although the slope indicated similar changing speed in three groups for foot stimuli, relatively larger change was revealed in long-term MA users, compared with short-term MA user and non-MA user for hand stimuli. As for the intercept, results of within-group test by analysis of covariance showed either short-term or long-term MA group was larger than non-MA group both for hand stimuli and foot stimuli, respectively (Table 4). Taken together, the results of Task 3 suggested that MA abuse would lead to a deficit in visuospatial ability, which correlates with the duration of MA use.

**Table 4:** RT at different orientation angles on both hand and foot laterality tasks, as well as the slopes and intercepts of linear regression equation on RT in two groups.

	RT (ms)							
	0°	60°	120°	180°	Slope (ms/°)	Intercept (ms)	R^2^	P
**Foot**								
MA−	1328 ± 24	1420 ± 15	1479 ± 21	1541 ± 29	1.16	1337.2	0.988	0.006
Short-term MA+	1475 ± 50	1540 ± 53	1625 ± 63	1683 ± 74	1.18	1474.3^a^	0.995	0.002
Long-term MA+	1520 ± 33	1559 ± 55	1607 ± 57	1696 ± 43	0.96	1509.1^a^	0.961	0.020
**Hand**								
MA−	1237 ± 32	1296 ± 32	1448 ± 38	1574 ± 37	1.94	1214.3	0.974	0.013
Short-term MA+	1495 ± 58	1552 ± 63	1629 ± 74	1781 ± 48	1.56	1474.0^b^	0.922	0.026
Long-term MA+	1475 ± 35	1518 ± 45	1671 ± 53	1868 ± 51	2.22^b^	1433.2^b^	0.935	0.033

^a^p < 0.05, short-term/long-term MA+ group vs. MA− group for foot stimulus;

^b^p < 0.05, short-term/long-term MA+ group vs. MA− group for hand stimulus.

## Discussion

Although there are plenty of publications with regard to negative effects of chronic MA abuse on psychological, daily life and neurocognitive functions^2–16^, knowledge is still limited about its effect on the spatial cognitive ability. The present study found that, in comparison with non-MA users, MA abusers had similar RT and accuracy in the Simple Reaction Task (Fig. 1), but considerably longer RT and lower accuracy in both the spatial orientation task (Fig. 2) and the mental rotation task (Fig. 3), clearly indicating that MA abuse may lead to deficit in both the visuospatial ability and the spatial orientation ability, rather than a general deficit in attention.

As recognized, spatial orientation advantage exists in healthy population. For the majority, leftward visual field advantage and leftward attentional bias present on horizontal surface^36–38^. Evidence suggests that abnormal or diseased conditions would lead this slight but stable leftward attentional bias disappeared or even inversed^37^. For instance, the disappearance of leftward attentional bias and a significant rightward in attention were observed in health subjects deprived of sleep for 24 hours^37^, which is quite likely associated with the circadian-related vigilant variations^38^. Interestingly, such phenomenon was also observed in patients with right hemispheric cerebral damage, who presented unilateral neglect^39^. It is well recognized that right hemispheric cerebrum plays an important role in spatial processing. Once damaged, such advantage would become weaken or even disappeared^40^. Spatial bias is linked to deficient alertness. Change of alertness level with shift in spatial attention may account for the rightward spatial bias, occasionally seen in adult neglect patient^41^. In this regard, special attention should be paid on those patients for their safety in daily life activity. In parallel, consideration should be taken into such abilities for those careers requiring highly developed spatial abilities or navigation in real and virtual environments (such as pilots, engineers, and architects).

In the present study, leftward attentional bias existed in non-MA abusers in the Spatial Orientation Task, who took shorter RT to judge the orientation of leftward foot pictures than that of rightward ones. On the contrary, such bias was not observed in MA abusers. Meanwhile, in comparison with non-MA users, MA abusers took longer RT to judge the orientation of leftward, but NOT rightward foot pictures (Fig. 2). Such phenomena quite likely relate to MA abuse-induced damage in cortical grey and white matter. During the Traditional Stroop Task, less activation was detected in MA abusers than control subjects in right-inferior-frontal gyrus, motor cortex, anterior-cingulate gyrus and anterior-insular cortex under incongruent condition^42^. Differences in task-related activity were also demonstrated in certain cortical regions when performing a facial affect matching task between MA-dependent and healthy participants, with the former exhibiting less activation in right hemisphere including the ventrolateral-prefrontal cortex, tempero-parietal junction, anterior/posterior-temporal cortex, as well as fusiform gyrus^43^. In addition to cortical regions in right hemisphere, white matter may also be damaged by MA abuse. After examining diffusion tensor measures (DTM) in frontal white matter and basal ganglia, lower fractional anisotropy (FA) was revealed in MA abusers in right frontal white matter, implying axonal injury^44^. It is therefore believed that MA abuse may mainly target the right hemisphere, leading to different performances on the horizontal surface observed in the present study.

Our findings also showed that all participants spent much shorter RT to judge the direction of upward hand pictures than downward pictures, clearly indicating an upward advantage on vertical surface (Fig. 2). Similarly, Lee *et al*.^45^ ever reported that human brain responds to the stimulus in the upper-left visual filed much more quickly than that in the lower-left field. In the daily life, upward hand pictures are relatively more often presented, which may be linked with the upward advantage observed in this work. Relative to downward hand pictures, people trends to be more familiar with upward hand pictures and therefore spends less time to respond. It has been confirmed that shorter time would be taken to respond those stimuli in accordance with body part positions and easy to keep with (familiar positions)^46,47^. Also participants performed faster and made fewer errors in the anatomically possible-body locations (familiar pictures) compared with the anatomically impossible locations on the Mental Rotation Task^34^. Additionally, RT to human picture depends on body location as well. For example, mental rotation time was much less with hands putting on their knees than with their fingers intertwined behind their backs^48^. Taken together, performance on the vertical surface largely depends on the familiarity of stimulus.

Mental rotation performance was also examined, during which the same set of human body picture was applied as the stimulus, to assess two different mental rotation strategies, namely, object-based strategy and egocentric-based strategy). For the former one, participants only have to rotate or translate the body/body part in imagery and observe two simultaneously presented pictures without changing their egocentrism. During the so called “the same-different task”, two pictures are judged as the same (non-mirror images) or not (mirror images), without considering their views and angular orientations. The latter one, on the other hand, is designed to assess the egocentric-based strategy, by judging which side a body part belongs to, the participant would instinctively imagine his/her body position in line with the stimulus picture^34,49^. It has been extensively reported that the mental rotation reaction time would change with angular disparity^34,50–53^. Consistently, our findings showed that RT increased progressively with the angle of stimulus picture increasing from 0° to 180°, and subsequently decreased with the angle further increasing from 180° to 300°, for both foot and hand stimuli (Fig. 3). Moreover, considerably poor visuospatial ability was confirmed in MA abusers, reflected by their performances in accuracy and RT at each orientation angle for either foot or hand stimuli. It was further supported by the longer intercept in MA abusers for either foot or hand stimuli and higher slope in long-term MA group (Table 4).

A linear relationship was revealed between RT and angular disparity within 0° to 180° in both groups, which is similar to Jansen’s study on the elderly^34^. Nevertheless, in Zacks’s study^32^, linear relationship was observed in an object-based same-different task, but not in an egocentric-based left-right mental rotation task. As a common sense, the imagination of rotating single hand stimulus involves physiological body movement, thus being regarded as one kind of egocentric-based mental rotation strategy^34,50–53^. However, evidence indicates that people sometimes tends to view a single hand picture as an object. Devlin *et al*.^54^ ever assessed the mental rotation ability using picture of alphanumeric character (5 or R), a single 3D photographed hand, or the whole-body as stimulus. For either younger or older adult, the regression line between RT and angular disparity fitted much better for alphanumeric character and single-hand stimulus, than for whole-body stimulus. Similar to the results of Devlin *et al*.^54^ and Parsons *et al*.^55^, our finding of a linear relationship in single hand/foot Mental Rotation Task, supports the idea that a single hand picture as an object is involved in object-based strategy. That is to say, the processing of single hand/foot mental rotation in this work may involve both the object-based and egocentric-based mental rotation strategies.

The inverse of the slope of regression line between RT and angular disparity gives an indication of mental rotation speed change^34,56^. The intercept of RT regression equation reflects the processes of perceptual comparison stage and decision stage on the Mental Rotation Task^34,57^. In comparison with non-MA users, long-term MA group had higher slope, indicating that MA abuse with longer duration had more negative effect on spatial process speed. Meanwhile, both long-term and short-term MA abusers exhibited larger intercept on the single hand and foot Mental Rotation Task, implying that MA abuse has negative impact in the perceptual comparison stages and the decision processes. Wang *et al*.^58^ ever investigated the effect of abstinent time on decision-making using the Iowa Gambling Task, and suggested that MA abuse has long-lasting influence on the decision-making performance for at least 3 months. More recently, brain activation was examined during the decision-making task with an aversive interoceptive challenge (anticipating and experiencing episodes of inspiratory breathing load) using functional magnetic resonance imaging. The poor decision-making performance in MA abuser was related to inadequate activation of many brain areas, including anterior insula (AI), inferior-frontal gyrus (IFG), posterior insula (PI) and anterior cingulate cortex (ACC)^59^. Thus, poor performance in the Mental Rotation Task observed in MA abusers, especially in long-term MA group, may be attributable to the deficits in the above-mentioned brain areas. Further investigations are warranted to prove this hypothesis.

In summary, our findings suggested that MA abuse may lead to a general deficit in the visuospatial ability and the spatial orientation ability. It may mainly target the right hemisphere, leading to a changing performance on the horizontal surface. As for the visuospatial ability, MA abuse has negative impact in the perceptual comparison stages and the decision processes. Moreover, both the cognitive speed and the accuracy were affected on the Mental Rotation Task, however, only the cognitive speed on the Spatial Orientation Task, which indicates that MA abuse may affect the abuser’s visuospatial ability more seriously than spatial orientation ability.

## Methods

### Participants

**Figure 4:**
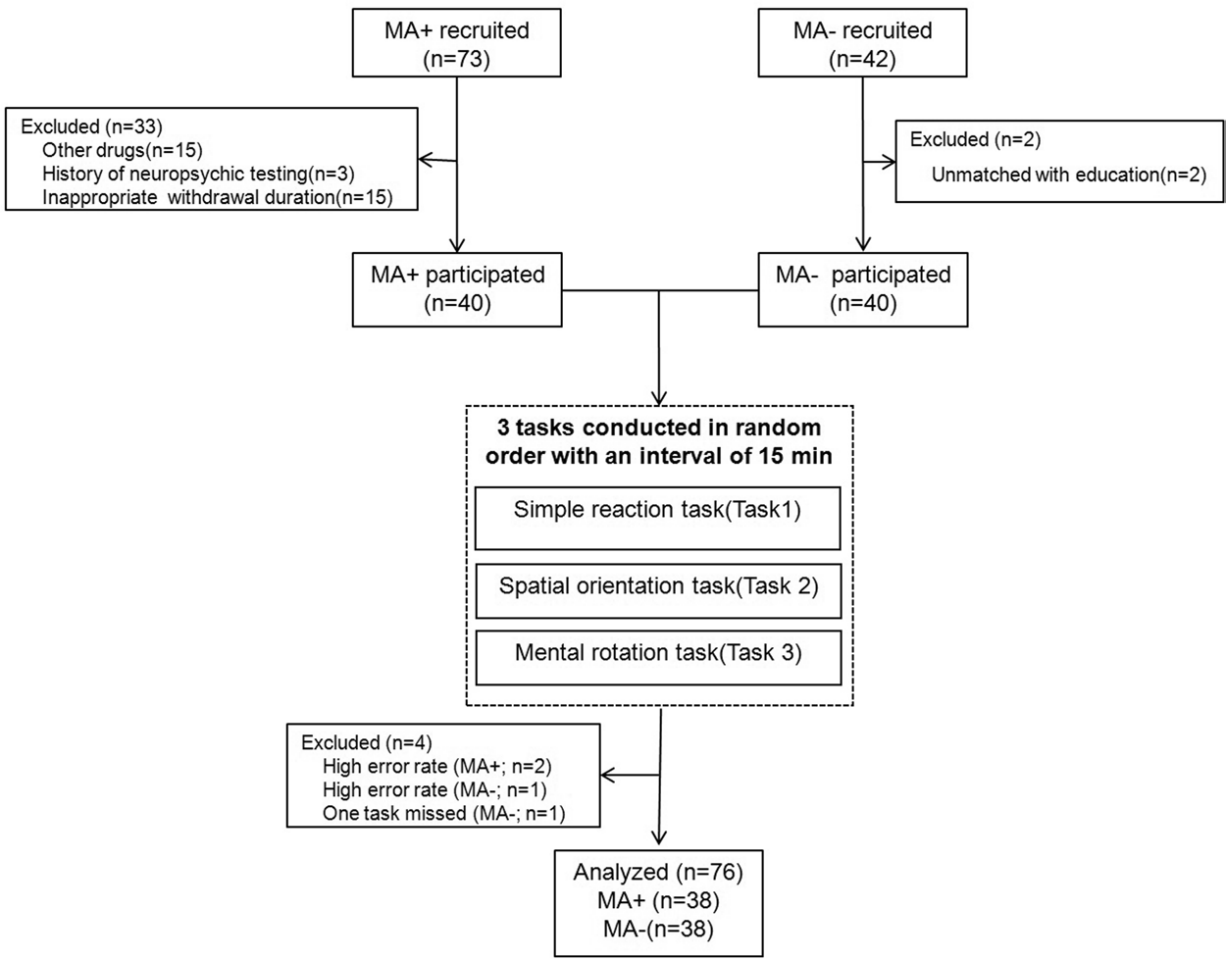
Research flowchart of this project.

Figure 4 illustrated the flowchart of this study. Briefly, a total of forty MA users (MA+ group, 20 males and 20 females) and 40 healthy non-drug users (MA− group, 20 males and 20 females) participated in this study. They were screened and recruited from Shanghai Compulsory Isolation Detox Center from September 2012 to April 2013. They were generally healthy by physical examination, medical history, and laboratory tests. The protocol was approved by the Ethics Committee of Capital Medical University, China. The complete details of the entire study design and procedures involved were in accordance with the Declaration of Helsinki. Written informed consent was obtained prior to participation.

MA+ subjects were included only if they fulfilled the following criteria: 1) were ≥18 years of age; 2) met DSM-IV-TR (Disorders listed in the Diagnostic and Statistical Manual of Mental Disorders—Forth edition, DSM-IV-TR American Psychiatric Association, 2000) criteria for a history of MA abuse with average use of at least 0.2 g/day, at least 2 days/week, for at least 6 months; 3) had been abstinent from MA use for about 14 days with the last consumption within 3 weeks before the experimental day; 4) provided a negative urine drug screen for cocaine, opiates, benzodiazepines, and cannabinoids; 5) did not have a history of abuse on other substances including alcohol, tobacco, cocaine, opiates, cannabis and barbiturates; and 6) were right-handed, and had normal vision or normal corrected visual acuity. MA− subjects also met all these criteria but a history of drug abuse. For both MA+ and MA− subjects, exclusion criteria included co-morbid or history of psychiatric illness, neurological disorder, or major chronic medical illnesses that may confound the results or analysis of this study; use of medications that affect the central nervous system; and pregnant.

MA+ subjects were evaluated by their drug use history and psychiatric condition during face-to-face interview. The influence of MA abuse on the subject’s life was assessed with the clinic institute withdrawal syndrome scale of craving, MMSE, and state-trait anxiety inventory (STAI). The psychiatric symptoms were assessed by the Symptom Checklist-90 (SCL-90) questionnaire.

### Experimental Procedure

**Figure 5:**
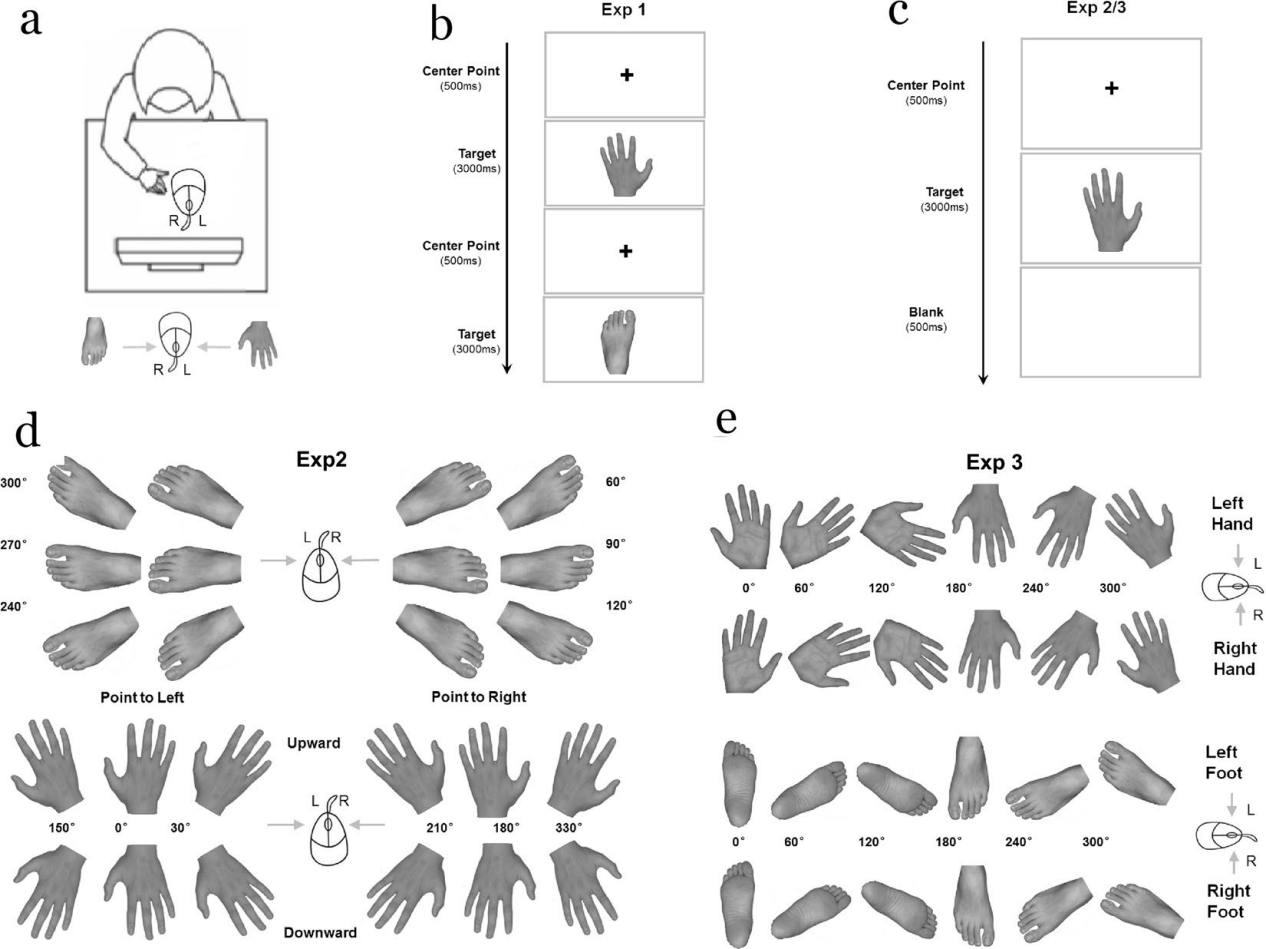
A diagram to illustrate the experimental procedures. Subjects were seated in the front of a computer screen to perform the Simple Reaction Task (Task 1), the Spatial Orientation Task (Task 2), and the Mental Rotation Task (Task 3) in a random order (**a**). During Task 1, each subject was asked to press the key of mouse as quickly and accurately as possible by the right hand (**b**). During Task 2 and 3 (**c**), each subject was instructed to press the left or right mouse button (left or right-click) according to either the direction of finger or toe during Task 2, or the laterality (left or right) of hand or foot during Task 3. Stimuli in Task 2 were presented in 2 different views and oriented in one of 6 clockwise orientation angles (0°, 30°, 150°, 180°, 210° and 330° for foot stimuli, as well as 60°, 90°, 120°, 240°, 270° and 300° for hand stimuli (**d**). As for Task 3, stimuli were presented in 2 different views and oriented in one of 6 clockwise orientation angles (0°, 60°, 120°, 180°, 240°, and 300°) in a random order (**e**).

All experiments were preformed in a soundproof, light-isolated chamber with temperature of 25 ± 1 °C. Subjects were seated in the front of a 17 inch computer screen (Lenovo ColorSync), 58 cm away from their eyes. Each stimulus, presented in the center of monitor with a width of 3.6~7.1 cm and a height of 5.1~7.1 cm, was controlled with E-Prime (Psychology Software Tools Inc., Pittsburgh, USA) (Fig. 5a).

As for each task, training trial identical to the formal one was initially conducted for 20 times. During Task 1, subjects were asked to focus on the center of monitor, and press the key of mouse as quickly and accurately as possible by the right hand (left key for hand picture and right key for foot picture) (Fig. 5b). During Task 2 and 3 (Fig. 5c), a fixation cross “+” was first presented in the middle of monitor for 500 ms, followed by a target stimulus (hand/foot picture) for 3000 ms. Each subject was instructed to keep his eye on the center of monitor and press the left or right mouse button (left or right-click) according to either the direction of finger or toe during Task 2, or the laterality (left or right) of hand or foot during Task 3^40^. Once the button was pressed, the stimulus on the screen would disappear immediately, followed by a blank interval of 500 ms.

### Materials

Each participant conducted 3 tasks in random order, namely, the Simple Reaction Task (Task 1), the Spatial Orientation Task (Task 2), and the Mental Rotation Task (Task 3), among which the last two tasks are commonly used to examine the spatial function^34,60–62^. Stimuli in Task 1 were two pictures, one upright hand and one upright foot. Nevertheless, stimuli in Task 2 and 3 consisted of 6 naturalistic color pictures of either hand or foot, respectively. The left hand or foot was actually mirror image of the original right one. During Task 2, stimuli were presented in 2 different views (dorsum, palm/plantar) and oriented in one of 6 clockwise orientation angles (0°, 30°, 150°, 180°, 210° and 330° for foot stimuli, as well as 60°, 90°, 120°, 240°, 270° and 300° for hands stimuli) (Fig. 5d). On the other hand, stimuli in Task 3 were presented in 2 different views (dorsum, palm/plantar) and oriented in one of 6 clockwise orientation angles (0°, 60°, 120°, 180°, 240°, and 300°) in a random order, with 0° defined as the upright orientation with the fingers/toes pointing upwards^35^ (Fig. 5e). A total of 258 stimuli were included in either Task 2 or Task 3.

### Data analysis

For each trial, the accuracy and RT were automatically recorded. Accuracy was defined as the number of correct response (in percentage) in relation to the original number of response, and RT as the time period from the stimulus onset to the participant’s response. Only those trials with RTs in the range of 300 ms and 3000 ms were included for further data analysis. Statistical analyses were performed using SigmaStat 3.5 (SAS Institute Inc., Cary, NC). Group differences were analyzed in demographics and clinical characteristics as well as experimental data using independent samples t tests or repeated two-way ANOVA (analysis of variance) where appropriate. Bonferroni t-test was conducted as post-hoc test to analyze differences between-subjects, and within-subjects (two-tailed). η2 (Eta squared) was calculated to show the effect size in ANOVA. Results were expressed as the mean ± standard error. Statistical significance was set at p < 0.05.
